# Aquaporin 5 (AQP5) expression in breast cancer and its clinicopathological characteristics

**DOI:** 10.1371/journal.pone.0270752

**Published:** 2023-01-27

**Authors:** Se Jin Jang, Chulso Moon

**Affiliations:** 1 Department of Pathology, Asan Medical Center, College of Medicine, Ulsan University, Seoul, Republic of Korea; 2 Department of Otolaryngology-Head and Neck Surgery, The Johns Hopkins Medical Institution, Cancer Research Building II, Baltimore, Maryland, United States of America; 3 HJM Cancer Research Foundation Corporation, Lutherville, Maryland, United States of America; Institute of Biomedicine of Seville-IBiS, SPAIN

## Abstract

The role of aquaporin water channels (AQPs) has become an area of great interest in human carcinogenesis. In this report, we have demonstrated the expression of AQP5 in breast cancer by analyzing 591 tissue samples with 7-year follow-ups. By immunochemistry analysis, AQP5 overexpression was observed in 36% (212/591 cases). Then, we have focused on the clinicopathologic variables among cancer tissue samples with strong AQP5 expression (3+ expression, 60/591 cases). The strong AQP5 expression was positively correlated with tumor grade in BCs (p<0.001) and was more frequent in ER/PR-negative BCs than positive ones (14.9% vs. 3.3% and 13.1% vs. 4.8%, respectively, both p<0.001), while Her2/neu-positive status was positively correlated with strong expression of AQP5 (p = 0.005). Of note, breast cancer patients with positive AQP expression (212/591 cases) showed a less favorable breast cancer specific survival rate over 7 years of follow and we further conclude that AQP5 expression is an independent molecular marker associated with worse clinical outcomes. By fluorescence *in situ* hybridization (FISH), we have identified evidence of gene amplification in 3 of 30 readable breast cancer and further conclude that, in breast cancer, at least some part of AQP5 overexpression is associated with an aberration in the genome level.

## Introduction

Aquaporins (AQPs) represent a family of transmembrane water channel proteins widely distributed in various tissues throughout the body. Since the discovery the protein and genomic structure in early 1990s [1, 2]. AQPs have been shown to be as a key player for both transcellular and transepithelial water movement [1–3]. Two regions of the channel are critical for AQP function. These regions are Asn-Pro-Ala or NPA and the aromatic-arginine region and that these NPA regions are connected each other making an hourglass model initially proposed by Agre et al. [1]. While a majority of AQPs are localized in the plasma membrane, some isoforms are present in the cytoplasmic compartments, particularly in the endoplasmic reticulum, where and their translocation to the plasma membrane is crucial in the regulation of water transfer [1–3], and may be involved in various process of cellular homeostasis. AQPs are expressed in many epithelial, endothelial and other tissues, with at least 13 AQPs being described in mammals with their genomic localizations [3–5]. Initial studies have classified at least two groups of AQP depending on their water transport and other transporter capabilities [1, 2]. Aquaporins AQP1, AQP2, AQP4, AQP5 and AQP8 are primarily water selective channels, while AQP3, AQP7, AQP9 and AQP10 (called “aqua-glyceroporins”) also transport glycerol and other small solutes in addition to water [1–6]. Since the initial description of AQP1 induction during erythrogenesis, genetic regulation of various AQPs has been studied. Additionally, while AQPs also can be modified by phosphorylation of various amino acids and its gating activity can be potentially modified depending on intracellular and extracellular environment including pH, oxygen, pressure, temperature, and solute gradient [2, 5–9]. Recently, new roles of AQPs have been characterized in terms of cellular proliferation and survival. For example, each AQP type plays a meaningful role in human carcinogenesis such as facilitating proliferation, migration, invasion, metastasis in addition to the drug resistance and potential prognostic markers in specific cancer type(s) [3–14]. Increased expression of AQP5 was initially reported in colon and pancreatic cancers [15–17]. As described above, *in situ* hybridization demonstrated that during colorectal carcinogenesis, the expression of AQPs 1 and 5 was induced in early-stage disease (early dysplasia) and maintained through the late stages of colon cancer development [15, 17]. These observations lead us to study molecular mechanisms behind AQP5 induced oncogenesis.

An overexpression of AQP5 in NIH3T3 cells and BEAS-2 cells have demonstrated a significant activation of the Ras pathways and that AQP5 mediated activation of Ras was shown to be mediated by phosphorylation of the cAMP-protein kinase (PKA) consensus site of AQP [4, 18, 19]. In fact, expression of AQP5 also seems to be regulated by PKA mediated phosphorylation of AQP5 [18]. Another study by Woo et al. has demonstrated that ectopic expression of human AQP5 (hAQP5) in BEAS-2 cells induces many phenotypic changes characteristic of transformation both *in vitro* and *in vivo* and this effects seem to be dependent upon the phosphorylation of a PKA consensus site located in a cytoplasmic loop of AQP5 [18, 19]. More importantly, hAQP5 overexpressing cells showed an increase in retinoblastoma protein phosphorylation through the formation of a nuclear complex with cyclin D1 and CDK4 [17]. These data had provided a unique molecular mechanism for colon cancer development through the interaction of hAQP5 with the Ras/extracellular signal-regulated kinase/retinoblastoma protein signaling pathway, extending the role of AQP5 expression on in the regulation of cell cycles. In the case of lung cancer, Chae et al. [19] has reported that, among more than 400 resected non-small cell lung cancer samples, various degrees of AQP5 expression haves been observed with significant prognostic implications. Likewise, other studies have shown that AQPs 1, 3 and 5 were expressed in various degrees among different type of resected non-small cell lung cancer (NSCLC) samples [20]. Importantly, we have developed AQP5 transgenic mouse and found that AQP5 increased tyrosine kinase activity among transgenic mice carrying human AQP5 expression constructs (manuscript submitted).

In terms of breast cancer (BC), initial study using BC tissue samples, expressions of AQPs 1, 3, 4, 5, 10, 11 and 12 were examined and various AQPs expressions demonstrated in human breast cancer (BC) with minimal expression in surrounding normal breast tissues [21]. AQP1 was expressed in cell membranes and its expression was higher in cancerous tissues as compared to accompanying normal tissues. On the contrary, AQP4 was expressed in the cell membrane and cytoplasm and was detected markedly stronger in normal than in cancer tissues. AQP5 was expressed mainly in cell membranes in carcinoma tissues, while it was absent in normal breast tissues [21]. A similar study has demonstrated a prominent AQP5 expression in breast cancer cells, while such expression was accompanied by the loss of polarity of ductal epithelial cells during the progression of breast carcinoma. Suppressed expression of AQP5 by the treatment with shRNA or hyperosmotic stress in MCF7 cells was associated with significantly reduced cell proliferation and migration [22]. Both of these reports suggested that AQP5 overexpression is likely to play a role in cell growth and metastasis of human breast cancer. Likewise, immunohistochemical analysis shows significant overexpression of AQP 5 in breast tumors from early breast cancer patients and was correlated with the disease prognosis particularly in patients with ER/PR+ tumors [23].

In this report, based on a large cohort of samples of 591 resected breast cancer samples, we have examined expression of AQP5. In this study, we have focused on patient groups with high level of AQP5 expression (60/591 cases) and examined their expression pattern and various clinicopathological characteristics like ER/PR or HER2 status. Also, based on 7 years of follow up data, we have studied prognostic implications of overall AQP5 expressions (212/591 cases). Finally, we have studied gene amplifications of AQP5 as one of the causes for strong expression of AQP5 in both lung and breast cancer tissue samples. We further conclude the at least some of groups with a strong AQP5 expression in breast cancer is likely to be driven by gene amplification as seen for HER2 gene among some group of breast cancer.

## Materials and methods

### Cancer tissue microarray

Tissue microarrays (TMAs) were constructed using formalin-fixed, paraffin-embedded tissues of 591 BC samples obtained from the Department of Pathology of Asan Medical Center under the approval of the Internal Review Board (IRB number: 2006–0019). To participate in the study, each patient has provided written consent with his or her own signature at the same time with signature from witness and such consent was provided as a part of protocol. All data were fully anonymized before they were accessed for this report. The breast samples were obtained from January 1993 and December 1998. The original H&E stained slides were reviewed by study pathologists and representative areas of each tumor were arrayed to the triplicated blocks to minimize the tissue loss and overcome the heterogeneity of the tumors. For lung cancer tissue microarray, it was previously described from Chae at al report [19]. Tissue microarrays (TMAs) were constructed using formalin-fixed, paraffin-embedded tissues of 419 NSCLC obtained from the Department of Pathology of Asan Medical Center under the approval of the Internal Review Board (IRB number: 2006–0019). The lung samples were obtained from 1996 to 1999. The original H&E stained slides were reviewed by study pathologists and representative areas of each tumor were arrayed to the triplicated blocks to minimize the tissue loss and overcome the heterogeneity of the tumors. Some of the samples from NSCLC tissue microarray was used as a positive control for AQP5 expression among breast cancer tissue microarray samples.

### Immunohistochemistry

The immunostaining procedures were performed using the Benchmark automatic immunostaining device (Ventana Medical System, Tucson, AZ, USA) with affinity-purified goat antibody raised against the 19-amino acid sequence (aa 251–269) of the COOH-terminus of human AQP5 (Alpha Diagnostic, San Antonio, Texas) at a 1:50 dilution. Tissue array sections (4 μm thick) were deparaffinized in xylene, rehydrated in graded alcohols, and treated with 3% hydrogen peroxide in methanol at room temperature for blocking of endogenous peroxidase activity. The AQP5 antibody was visualized using the avidin-biotin-peroxidase technique (DAKO LSAB kit; DAKO Cytomation, Carpinteria, CA) and followed by chromogen detection with diaminobenzidine (DAB). Negative controls were performed by omitting the primary antibody incubation step.

### Scoring of immunohistochemistry

Immunostaining on the TMAs was graded semi-quantitatively considering both staining intensity and percentage of positive tumor cells by two study pathologists (SJJ and MJK) blinded to the clinicopathologic variables. The staining intensity was arbitrarily scored on a scale of four grades: 0, no staining of cancer cells; 1, weak staining; 2 moderate staining; 3, strong staining. The percentage of stained tumor cells was graded on a scale of 2 grades: 0, <10% and 1, >10%. AQP5 expression in the cancer tissue was defined as positive when the product of intensity score by percentage score is 1 or more. Both histological type and grade were confirmed on hematoxylin and eosin (H&E) stained TMA slides.

### Fluorescence *in situ* hybridization (FISH) analysis

Sixty BCs which overexpressed AQP5 (score = 2 or 3) were reassembled in two TMA blocks for fluorescence *in situ* hybridization (FISH) analysis, each. Sections (5 μm thick) of the TMA slides were deparaffinized and pretreated for FISH. The probe for AQP5 detection was derived from Homo sapiens 12 BAC RP11-469H8 containing the whole AQP5 gene (GenBank Accession No. AC025154), and labeled with rhodamine (Macrogen, Seoul, Korea). Two genomic DNA clones with 91 kb and 68 kb sizes franking 12p13.31 region labeled with FITC were used as control probes (Macrogen, Seoul, Korea). FISH was performed using Vysis reagents according to the manufacturer’s protocols (Vysis, IL, USA). Slides were counterstained with 4, 6-diamidino 2-phenylindole for microscopy. The signal was counted under ×1000 magnification.

### Statistical analysis

Statistical analyses were performed using the chi-square test with SPSS software (SPSS 12.0K Inc., Chicago, Illinois, USA). The patients’ mean age and tumor size were evaluated by t-test. Survival curves were produced using the Cox proportional hazards model to test the risk of cancer death. Prognostic factors were examined by both univariate and multivariate analyses. All p-values were two-sided, and differences at p<0.05 were considered statistically significant.

## Results

### Tissue microarray and clinicopathological correlation of AQP5 expression

**Fig 1:**
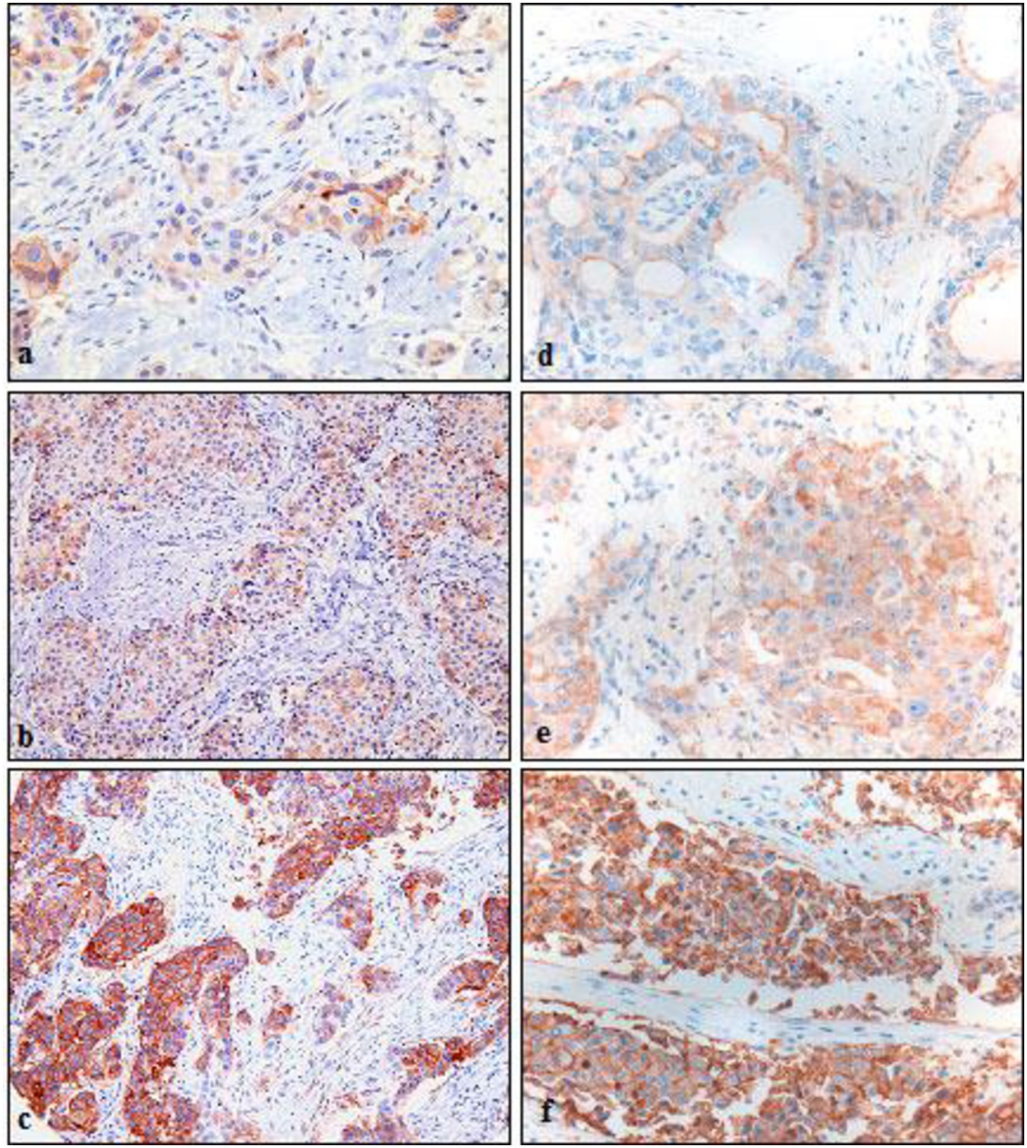
Immunohistochemistry of resected breast cancer tissue samples for the detection of AQP5 expression. Photomicrographs of AQP5 immunostaining in arrayed breast cancer tissues are shown. Non-small cell lung cancer (NSCLC) are presented as a comparison. Examples of weak (a and d,1+), moderate (b and e, 2+), and strong (c and f, 3+) immunoreactivity in breast cancers (d to f, respectively) and NSCLCs (a to c, respectively). Original magnification X200.

To further investigate the significance of our previous *in vitro* and *in vivo* observations, we have initiated our study by preparing tissue micro array. The study for clinical implication of AQP5 expression in breast cancer was based on analyzing a TMA containing 591 BCs. Immunohistochemistry of resected breast cancer tissue samples for the detection of AQP5 expression was performed at the same time with tissue samples from NSCLC as control. We have classified expression of AQP5 into three categories. Examples of weak staining (1+, weak expression), moderate staining (2+, moderate expression), and strong staining (3+, strong expression) immunoreactivity in breast cancers (Fig 1d–1f, respectively) and NSCLCs (Fig 1a–1c, respectively) are presented. By immunochemistry, overall, AQP5 expression was observed in 36% (212/591 cases). Based on the expression pattern from tissue microarray, we have focused on the clinicopathologic variables among cancer tissue samples with strong AQP5 expression (3+ expression, 60/591 cases) (Table 1). The strong AQP5 expression was positively correlated with tumor grade in BCs (p<0.001). Interestingly, among tissue samples from strong AQP5 expression, a strong AQP5 expression was more frequent in ER/PR-negative BCs than positive ones (14.9% vs. 3.3% and 13.1% vs. 4.8%, respectively, both p<0.001), while Her2/neu-positive status was positively correlated with a strong AQP5 expression (p = 0.005). Of note, among 591 patients, 7 patients were male breast cancer patients. However, this number is small and proper interpretation of clinicopathological characteristics based on gender difference is not feasible (Table 1).

**Table 1:** Demographics of the breast cancer patients with strong AQP5 expression and tumor characteristics.

Clinical findings		Breast cancer (strong AQP5 Expression/cases) (AQP(+)/n = 60/591)	*p*-value*
Mean Age (range, yrs)		47.2 (24–88)	0.238
Gender	Woman	60/584	0.664
	Man	0/7	
Histologic subtype		IDC, NOS (56/532)	0.395
		Invasive MC (3/21)	
		ILC (0/13)	
		Others (1/25)^$^	
Grade^^^ WD (HG 1)	WD (HG 1)	0/70	0.006
	MD (HG 2)	28/278	
	PD (HG 3)	32/243	
Mean tumor size (cm)		3.1	0.841
T stage	T1	19/191	0.294
	T2	37/323	
	T3	2/60	
	T4	2/17	
	Unknown	0	
N stage	N0	23/263	0.668
	N1	18/151	
	N2	8/73	
	N3	11/95	
	Unknown	0/9	
TNM stage I	I	13/128	0.305
	II	27/282	
	III	19/179	
	IV	1/2	
	**Unknown**	0	
Immunophenotypes			
ER (+)		8/243	0.000
ER (-)		52/348	
PR (+)		10/210	0.001
PR (-)		50/381	
Her2/neu (+)		25/156	0.005
Her2/neu (-)		35/435	

*Pearson *X*^2^ test of cross table between AQP5 positive and negative groups except for age and tumor size calculated by student t-test.

^$^Other subtypes include 5 mucinous, 7 medullary, 6 metaplastic, 2 tubular, one papillary, and 2 cribriform carcinomas.

^Breast cancers were accessed using modified Bloom-Richardson classification.

### Expression of AQP5 as a predictive marker for survival

**Fig 2:**
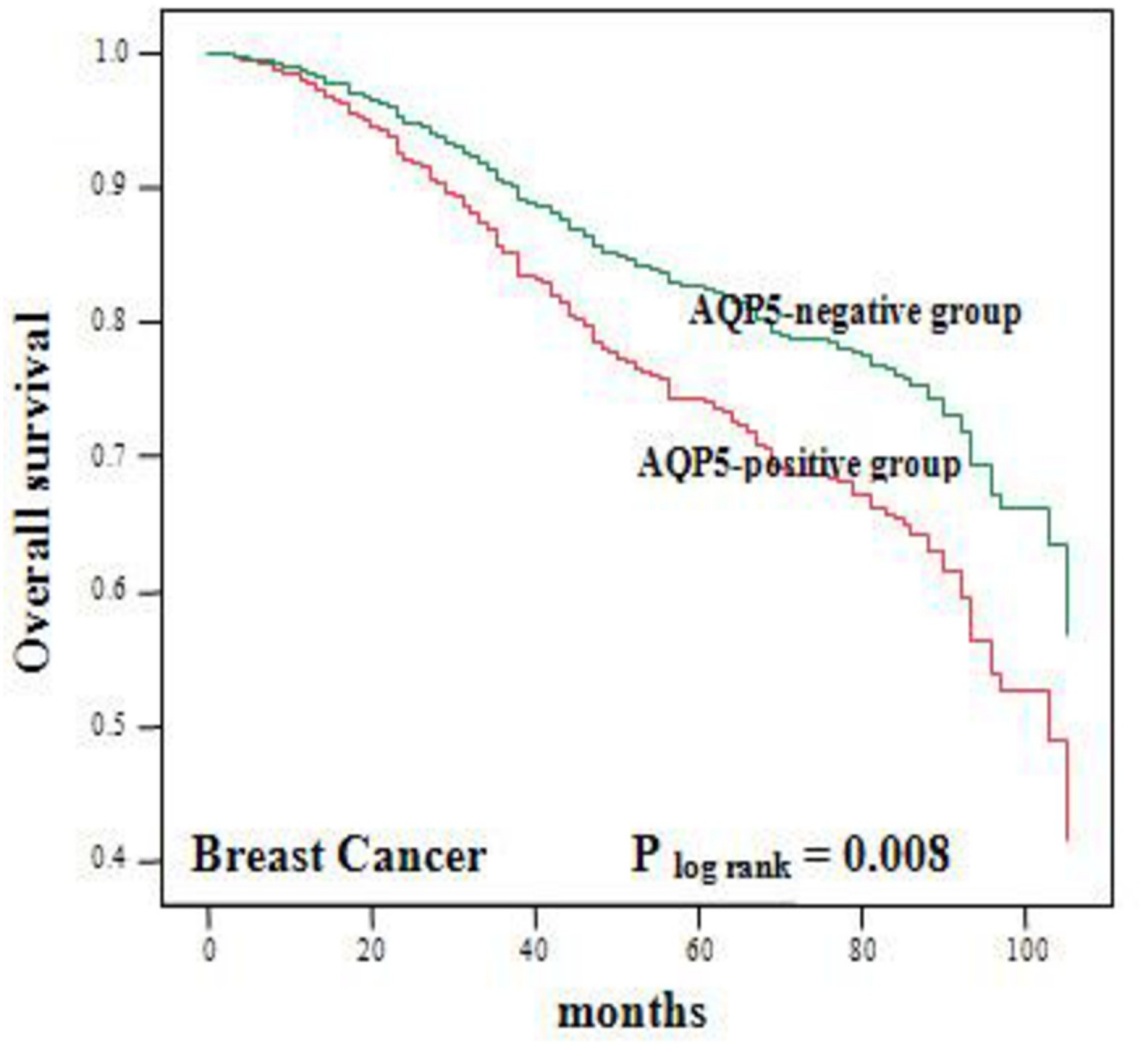
Prognostic implications of AQP5 expression. To ascertain the clinical significance of AQP5-positive status, the relationship between AQP5 status and patients’ survival rates is analyzed. Remarkably, AQP5-positive cases showed a worse breast cancer specific survival rate (p = 0.008).

We have analyzed the prognostic implications of AQP5 expression among 591 patients and we have followed patient’s overall survival up to 7 years of diagnosis (Fig 2). In this analysis, patients’ groups with positive AQP5 expression carried (212 cases) a less favorable breast cancer specific survival rate (p = 0.008). Moreover, by multivariate analysis adjusting histologic grade and tumor stage, AQP5 overexpression (212 cases) in breast cancer is correlated with a poorer disease specific survival rate (p = 0.035; RR = 1.567; 95% CI 1.032–2.375). AQP5 status therefore is an independent molecular marker associated with worse clinical outcomes in BC.

### Gene amplifications of AQP5

**Fig 3:**
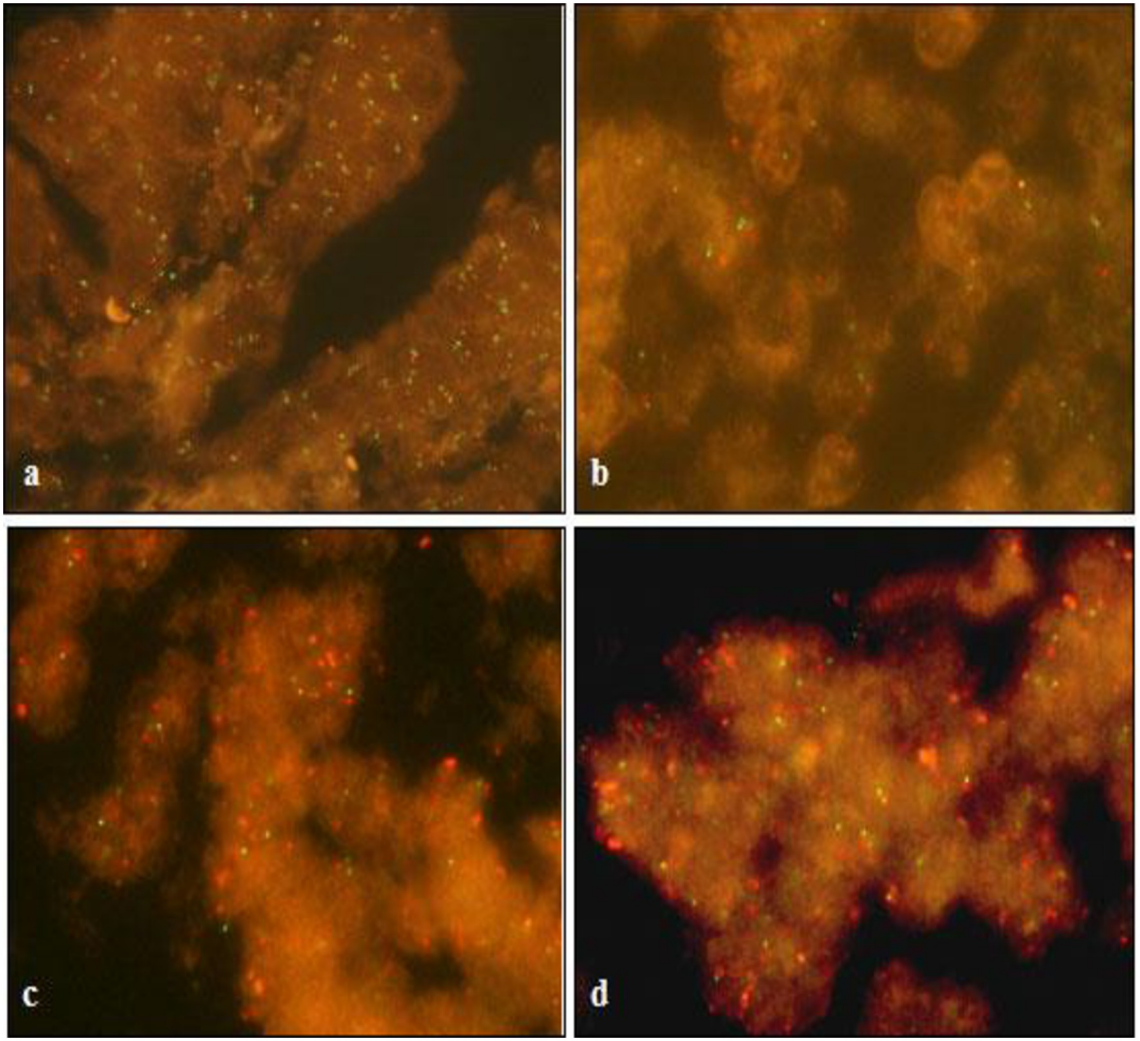
FISH analysis for resected tissue samples from NSCLC and breast cancer patients. In the FISH analysis, no gene amplification is identified in any of 60 NSCLCs tested, and one representative example is shown (a). In breast cancers, however, at least three of 60 cases showed evidence of gene amplification. Representative examples of negative (b) and positive cases (c and d) (green color: FITC labeled control probe, red color: rhodamin labeled AQP5 probe). Original magnification ×1000. AQP, aquaporin water channel; FISH, fluorescence *in situ* hybridization; NSCLC, non-small cell lung cancer.

To elucidate the mechanisms of AQP5 overexpression, by FISH we have identified evidence of gene amplification in 3 of 30 readable BC cases (Fig 3), and further conclude that, unlike AQP1, at least some part of AQP5 overexpression is associated with an aberration in the genome level [19]. To screen gene amplification status, we reassembled the BC tissues showing AQP5 score 3 and some of score 2 into 2 TMA blocks and performed FISH.

## Discussion

Breast cancer is a common malignant tumor that is affecting women with an increasing rate of incidence in many countries while it remains the leading cause of tumor-associated mortality in women worldwide [21–23]. Although several prognostic criteria have already been introduced to assist management after curative surgery for early breast cancer, the need for molecular markers has always been strongly suggested to discriminate individual variability and thus predict relapse or survival in patients with a similar clinical status, Estrogen, acting through predominantly ER), has a significant detrimental impact during its pathogenesis [24]. This forms the basis for endocrine therapy, with the application of pharmacological antagonists generally termed selective estrogen receptor modulators, such as tamoxifen. These have resulted in significant improvements in quality of life as well as improved prognosis in a significant proportion of patients with clinically defined ER+ status [24–27]. In the last 10 years, with following the initial report from our group for on the role of AQP during colorectal development, several tumor cell types were shown to express AQPs *in vivo* in humans and rodents. Furthermore, prognostic implications have been established based on AQP expression in lung cancer, colon cancer, brain tumors and human glioblastoma [5–7, 9, 10, 15, 17, 19]. Presently, there is no clinical data so far which confirms the use of AQPs as diagnostic markers for breast cancer. However, many reports suggest a strong correlation between the expression profile of certain types of AQPs and breast cancer pathogenesis and prognosis [21–23, 28–33].

In this report, we have analyzed the largest number of breast cancer samples in studying expression of AQPs (Fig 1) with longest follow up (Fig 2). Overall, our report shows similar trend to other report between AQP5 expression and its impact on aggressiveness of cancer behavior and prognosis, while some of the details of findings show noticeable differences. For example, an initial report by Jung et al. [22] has, based on 20 samples, demonstrated that AQP5 expression was significantly higher among invasive carcinoma with lymph node (LN) metastasis to those without LN metastasis, while in our analysis, one of the key findings was the observation that tumor grade was positively correlated with high level of AQP5 expression. Likewise, the same group, in their subsequent study [23], analyzed AQP5 expression among 447 early-stage breast cancer patients and reported that 59.7% patients were identified as AQP5 over expression (positive), while in our analysis based on 591 breast cancer patients among all different stages, AQP5 overexpression was observed in 36% of analyzed patients.

In this report, we have run a detailed clinical pathological impact of patients (all stages) with strong AQP5 expression (3+ expression) (Table 1) and examined correlation between ER/PR/HER2 to the expression of AQP5. The strong expression of AQP5 was more frequent in ER/PR-negative group, whereas Her2/neu-positive status was positively correlated with the strong expression of AQP5. However, Jung et al.’s study did not show same correlation among early-stage breast cancer patients.

Of note, in report by Lee et al. [23], in a univariate analysis. AQP5 overexpression was significantly associated with survival for the selective patient’s group with ER/PR-positive and (HER2)-overexpression. By contrast, in our report, among 591 patients, which included early stage, intermediate stage and late stage, from 7 years of follow up, patients’ groups with positive AQP5 expression carried (212 cases) a less favorable breast cancer specific survival rate (Fig 2). Likewise, in Jung et al.’s report, a multivariate survival analysis revealed that AQP5 overexpression was an independent prognostic marker of survival for the early-stage breast cancer patients, a majority which are ER/PR positive. In our report, multivariate analysis adjusting histologic grade and tumor stage, AQP5 expression status among all stages of BC patients seems to be an independent molecular marker associated with worse clinical outcomes and this observation seems to be like Lee et al.’s [23] analysis among early-stage breast cancer patients. In summary, the similarity of our report to 3 prior reports [21–23] overall suggests that AQP5 expression carries a poor prognostic implication while some of the clinicopathological relationship between AQP5 expression with other key clinicopathological features like relationship between AQP5 expression and ER/PRE/HER and tumor grade seems to be different. These discrepancies are likely due to difference in evaluating AQP5 expression and groups of samples analyzed (early-stage vs all stages).

Of note, among 591 patients, 7 patients were male breast cancer patients, all of which breast cancer did not have a strong AQP5 expression. While this finding may carry potential different role of AQP5 during breast cancer tumorigenesis between female and male patients, the number of male cases is very small and interpretation for the clinicopathological characteristics based on gender difference is not appropriate (Table 1) and therefore may warrant appropriate future studies.

One key difference from our report to other prior reports is the fact that in our report, we have analyzed gene amplifications as one of the potential mechanisms underlying high degree of AQP5 expression. Since the genetic regulatory element and mechanisms of AQP1 induction during erythroid differentiation, genetic regulations of AQPs are still in their early stage of research endeavors [34, 35]. We have analyzed lung cancer tissues samples as a control and while we did not notice any degree of AQP5 gene amplification from lung cancer tissue samples, over 10 percent of small sized samples with AQP5 expression, we have confirmed gene amplification (Fig 3). Moreover, as discussed above, the fact that a strong expression of AQP5 and HER2 positivity is positively correlated might suggest that some group of BCs which carry HER2 positive, high AQP5 breast cancer, the gene amplification of AQP5 might be suggested as a key driving force for oncogenesis if our findings of AQP5 gene amplification is reproduced in a large number of breast cancer samples. De novo resistance to hormonal therapy occurs in about 30%–40% of patients and almost all initially responsive patients with late-stage metastatic disease eventually relapse due to treatment resistance. These forms of endocrine resistance invariably led to a more aggressive form of resurgent disease [27], and occur in parallel with cellular transition from epithelial to mesenchymal phenotype (EMT). Our findings for inverse correlation between AQP5 expression and ER/PR status, with the presence of gene amplifications of AQP5, may suggest a possible role of AQP5 targeted therapeutics for BC patients with hormone treatment resistances. Likewise, targeting AQP5 together with targeting HER2 for BC patients with AQP5/HER2 strongly positive group may provide a potential therapeutic opportunity, considering the accumulated success of Herceptin in managing HER 2 strong positive breast cancers [36–38]. In fact, Park et al. has recently reported identification of AQP5-regulating miRNAs, which could be exploited for the inhibition of breast cancer cell migration via the exosome-mediated delivery [39]. Also, we have initiated designing anti AQP5 therapeutic antibody and recently developed 4 novel therapeutic small molecules and have been tested various cancer cell lines with *in vitro* therapeutic efficacy. We envision that an anti AQP5 therapeutic antibody or small molecular inhibitors targeted to AQP5 expression among BC patients with strong AQP5 expression or genomic implications may open door to new levels of anti-breast cancer therapeutics [40].

## Supporting information

S1 File We have developed AQP5 transgenic mouse and found that AQP5 increased tyrosine kinase activity: Submitted manuscript file is attached. (PDF)
