# Supplementary material for: Aquaporin 5 (AQP5) expression in breast cancer and its clinicopathological characteristics
Source: PLoS One. 2023 Jan 27;18(1):e0270752. doi: 10.1371/journal.pone.0270752 (PMC9882752; doi:10.1371/journal.pone.0270752)
Supplement: S1 File — (PDF) [file pone.0270752.s001.pdf]

Aquaporin 5 (AQP5) promotes tyrosine kinase activity

Sung Koo Kang [a,b], Chul So Moon [a,b,*]

[a] *Department of Otolaryngology-Head and Neck Surgery, The Johns Hopkins Medical Institution, Cancer Research Building II, 5M3, 1550 Orleans Street, Baltimore, MD, 21205, USA*

[b] *HJM Cancer Research Foundation Corporation, 10606 Candlewick Road, Lutherville, MD, 21093, USA*

* Corresponding author.

E-mail address: csmoon6@gmail.com (C.S. Moon).

Abstract

The role of aquaporin water channels (AQPs) has become an area of great interest in human carcinogenesis. We developed AQP5 transgenic mice and demonstrated overall cellular hyperplasia due to over-expression of human AQP5, which also lead to dose dependent activation of Raf, Erk and Akt. Moreover, AQP5 increased tyrosine kinase activity in the new born mice and several organs from 6 months old mice, especially from colon and lung. Furthermore, by using recombinant human AQP5 protein, which was amplified and purified by using known bacuolar virus system, we have demonstrated that wild type AQP5 can carry a tyrosine kinase activity while two of its mutants cannot. Moreover, a potential synergistic augmented kinase activity was observed when AQP5 and epidermal growth factor receptor (EGFR) are combined. While some of findings are still preliminary, the data we present here may provide a biological rationale for designing novel anti AQP5 therapeutics.

Keywords: AQP5, EGFR, Tyrosine Kinase, Transgenic Mice

1. Introduction

Aquaporins (AQPs) represent a family of transmembrane water channel proteins widely distributed in various tissues throughout the body and AQPs have been shown to be as a key player for both transcellular and transepithelial water movement [1-3]. Two regions of the channel critical for AQP function, Asn-Pro-Ala or NPA, are connected each other making an hourglass model [1-6]. While a majority of AQPs are localized in the plasma membrane, some isoforms are present in the endoplasmic reticulum, where and their translocation to the plasma membrane is crucial in the regulation of water transfer [5,6], and may be involved in various process of cellular homeostasis. Being expressed in many epithelial, endothelial and other tissues, at least 13 AQPs were described in mammals with their genomic localizations [3-5]. So far, at least two groups of AQP depending on their water transport and other transporter capabilities [3,5,6]. Aquaporins AQP1, AQP2, AQP4, AQP5 and AQP8 are primarily water selective channels, while AQP3, AQP7, AQP9 and AQP10 (called "aqua-glyceroporins") also transport glycerol and other small solutes in addition to water [5,6]. In the functional level, AQPs can be modified by phosphorylation of various amino acids, while its gating activity also can be potentially changed depending on various environment including pH, oxygen, pressure, temperature, solute gradient [6-10].

Recently, new roles of AQPs have been characterized in terms of cellular proliferation and survival. For example, each AQP type plays a meaningful role in human carcinogenesis such as facilitating proliferation, migration, invasion, metastasis in addition to the drug resistance and potential prognostic markers in specific cancer type(s) [3,5,9-15]. Increased expression of AQP5 was initially reported in colon and pancreatic cancers [7,16,17]. As described above, *in situ* hybridization demonstrated that during colorectal carcinogenesis, the expression of AQPs 1 and 5 was induced in early-stage disease (early dysplasia) and maintained through the late stages of colon

cancer development [7,17]. These observations lead us to study molecular mechanisms behind AQP5 induced oncogenesis. Importantly, an overexpression of AQP5 in NIH3T3 cells demonstrated a significant activation of the Ras pathways and that AQP5 mediated activation of Ras was shown to be mediated by phosphorylation of the PKA consensus site of AQP5 [4,18] and these effects from AQP5 also seems to be regulated by PKA mediated phosphorylation of AQP5 [17-19]. Likewise, ectopic expression of human AQP5 (hAQP5) in BEAS-2B cells induces many phenotypic changes characteristic of transformation both *in vitro* and *in vivo*. Furthermore, the cell proliferative ability of AQP5 appears to be dependent upon the phosphorylation of a cAMP-protein kinase (PKA) consensus site located in a cytoplasmic loop of AQP5 [18,19]. Both findings from NIH3T3 cells and BEAS-2B cells indicate that hAQP5 plays an important role in human carcinogenesis through "PKA dependent phosphorylation of AQP5", which can activate Ras signaling pathways. Overexpression of wild-type hAQP5 increased proliferation and phosphorylation of extracellular signal-regulated kinase-1/2 in HCT116 colon cancer cells [18]. More importantly, hAQP5-overexpressing cells showed an increase in retinoblastoma protein phosphorylation through the formation of a nuclear complex with cyclin D1 and CDK4. These data had provided a unique molecular mechanism for colon cancer development through the interaction of hAQP5 with the Ras/extracellular signal-regulated kinase/retinoblastoma protein signaling pathway, extending the role of AQP5 expression on in the regulation of cell cycles. In the case of lung cancer, Chae et al. [19] has reported that, among more than 400 resected non-small cell lung cancer samples, various degrees of AQP5 expression haves been observed with significant prognostic implications. *In vitro* invasion assays using BEAS-2B cells stably transfected with various AQP5 expression constructs (wild type and two mutants, N185D (this mutant compromise proper membrane expression of AQP5) or S156A (This mutant compromise

PKA dependent phosphorylation) demonstrated that wild type AQP5 expression can induce cell invasions.

In this report, we have developed AQP5 transgenic mouse to test the impact of human AQP5 on mice pathophysiology. We here report cellular hyperplasia induced by human AQP5 and induced phosphorylation of downstream signaling. Finally, we provide some preliminary evidence for AQP5 as potential tyrosine kinase. We were interested in synergistic activation of epidermal growth factor receptor EGFR and Src tyrosine kinase when they were combined with purified human AQP5 proteins and demonstrate an augmented kinase activity between AQP5 and epidermal growth factor receptor (EGFR). As discussed above, AQP5 is known to activate multiple tyrosine kinases including Src and Ras with resulting activation of Raf, Erk and Akt. Therefore, we propose that AQP5 might carry a novel tyrosine kinase activity through which it can activate multiple downstream pathways and coordinate with EGFR during human carcinogenesis.

2. Materials and methods

2.1. Generation of transgenic mice and tyrosine kinase assay

To generate the expression vector, a 798-bp fragment containing the entire open-reading frame of the human AQP5 cDNA was cloned into pcDNA 3.1 containing ubiquitous CMV promoter. The construct was linearized at the ScaI site and submitted to the Transgenic Mouse Core Facility at the Johns Hopkins University School of Medicine for microinjection into B6SJLF1 mouse eggs. Transgenic mice were identified by polymerase chain reaction (PCR) amplification of a 798 bp product and a 516 bp product from genomic DNA extracted from tail biopsies using the following primers: AQP5 5' primer: 5'-ATGAAGAAGGAGGTGTGCTC-3', 3' primer: 5'-TCAGCGGGTGGTCAGCTCCATG-3', Internal Sequence 5' primer: T7 promoter/priming

sequence in construct, 3' primer: 5'-GCTGGAAGGTCAGAATCAGC-3', respectively. Five transgenic founder animals were identified and germ-line transmission of the transgene was demonstrated for the founders. Lines of the founder transgenic mice were established by crossing each founder mouse with an inbred strain to obtain the F1 generation, and the transgenic lines were propagated by breeding the littermates. AQP5 transgenic lines were housed in a specific pathogen-free environment. The transgenic expression and the observed phenotypes were stable for the following generations. Non-transgenic littermates were used for controls. All animal experiments were approved by the Central Animal Facility at the Johns Hopkins Medical Institute. For analysis of transgenic expression and signal proteins in AQP5-transgenic mouse, lungs, livers, kidneys, and salivary glands were removed under general anesthesia and polyadenylylated RNA and genomic DNA were isolated (TRIzol reagent, Invitrogen and Tissue DNA extraction kit, Qiagen) 6 months after mice were born. RNA were reverse-transcribed with the Moloney murine leukemia virus reverse transcriptase for 60 min at 37°C and the resulting cDNA or genomic DNA was PCR amplified with Taq DNA polymerase (Invitrogen) and above-mentioned primers and GAPDH primers. Protein extracts were prepared by grinding tissues on dry ice and then lysing them in the above-mentioned NP-40 lysis buffer. Signal proteins were detected by immunoblotting of protein extracts. Tyrosine kinase activity was measured as recommended by manufacturer (Sigma, Perkin Elmer). Two sets of assays were performed. Whole mice, 2 days after birth and each organ from 6 months old transgenic mice were studied for their kinase activity as compared to control.

2.2. Immunoblotting

Lysates from cultured cells and tissues were prepared in ice-cold NP-40 lysis buffer (10 mM Tris-Cl (pH 7.4), 137 mM NaCl, 10% glycerol and 0.1% Nonidet P-40) containing an inhibitor cocktail of 10 mM β-glycerol phosphate, 1 mM phenylmethylsulfonyl fluoride, 10 mM NaF, 10

mM Na orthovanadate, 4.5 U/mL aprotinin (Sigma), and 1 μg/mL leupeptin (Sigma). Crude protein lysates (25 μg) were separated by 4–12% SDS-PAGE (Invitrogen), transferred to nitrocellulose membranes (BIORAD), and blocked for 1 hour with 5% nonfat dry milk in Tris-buffered saline with 0.05% Tween-20. The following commercial antibodies were used for Western blot analysis: anti-FLAG (Sigma), anti-AQP5 (Alpha Diagnostic), anti-ERK (Santa Cruz Biotech), anti-phospho-ERK (Santa Cruz Biotech), anti phospho-RAF (Cell Signaling), anti-Akt (Cell Signaling), anti-phospho-AKT (Cell Signaling) and mouse anti-beta actin antibody (Sigma), anti-phospho-EGFR and anti-EGFR (Cell Signaling). Appropriate anti-rabbit and anti-mouse horseradish peroxidase-conjugated secondary antibodies (Amersham) were used. Immunoreactive bands were detected by enhanced chemiluminescence (Pierce).

2.3. Immunohistochemistry and immunofluorescence

The immunostaining procedures were performed using the Benchmark automatic immunostaining device (Ventana Medical System, Tucson, AZ, USA) with affinity-purified goat antibody raised against the 19-amino acid sequence (aa 251–269) of the COOH-terminus of human AQP5 (Alpha Diagnostic, San Antonio, Texas) at a 1:50 dilution. Tissue array sections (4 μm thick) were deparaffinized in xylene, rehydrated in graded alcohols, and treated with 3% hydrogen peroxide in methanol at room temperature for blocking of endogenous peroxidase activity. The AQP5 antibody was visualized using the avidin-biotin-peroxidase technique (DAKO LSAB kit; DAKO Cytomation, Carpinteria, CA) and followed by chromogen detection with diaminobenzidine (DAB). Negative controls were performed by omitting the primary antibody incubation step.

2.4. Generation of recombinant baculoviruses

The flag-tagged human wild type AQP5 wild type, S156A mutant and N185D [4,20] were

directly transferred into the baculovirus genome *in vitro* using BaculoDirect™ baculovirus expression system (Thermo Fisher). The *Escherichia coli* cell line DH10BAC, which carries the baculovirus genome cloned into a bacterial artificial chromosome (BAC), was used to introduce flag tagged N185D mutant gene using transposition methods according to the manufacturer's protocol (Invitrogen) [20]. PCR analysis of the bacmid DNA was used to confirm the introduction of the AQP5 gene into the baculovirus, and positive bacmid clones were transfected into low-passage Sf9 cells as described by o reconstitute infectious virus.

2.5. Baculovirus transfection and amplification

Sf9 cells (9×10^5 cells in 35-mm dishes) were transfected with bacmid DNA using Cellfectin reagent (Invitrogen) as previously described [20]. Generally, two independent bacmid clones were transfected for each expression construct. The supernatant of the transfected Sf9 cells, which contained the recombinant virus, was harvested 72 h after transfection, clarified, and designated as the P1 stock. Viruses were amplified in low-passage Sf9 cells. P2 and P3 stocks were typically 10^8 PFU/mL.

2.6. Purification of recombinant AQP5 and measurement of kinase activity

ProBond™ Nickel-Chelating Resin is used for purification of recombinant AQP5 proteins expressed in insect mammalian cells under native condition according to the manufacturer's protocol (Invitrogen). Tyrosine kinase activities were measured as followed by manufacture instruction (Sigma, Perkin Elmer).

2.7 Cell Culture and transfection

The Chinese Hamster Ovary (CHO) cell line was cultured in CHO media (Gibco) was supplemented with 10% fetal bovine serum and antibiotics (Gibco) at 37°C in a humidified environment containing 5% CO2. Different amount of AQP5-Flag expression construct and

EGFR expression construct, pCMV-EGFR, (Signosis) were transfected into CHO cells with FuGENE 6 (Roche, Switzerland) according to manufacturer's recommendations [19-20].

3. Result

3.1. Generation of transgenic mice

Five transgenic founder animals were identified carrying wild type human AQP5 expression construct and germ-line transmission of the transgene was demonstrated for the founders. Out of these 5 founder mice, three lines of transgenic mice were maintained and, depending on transgene copy number, transgenic mice were classified into three groups. In Fig. 1A, PCR product from mice carrying low copy, medium copy and high copy number of human AQP5 genomic DNA (DNA) and complementary DNA (cDNA) were shown with GAPDH expression as internal control.

3.2. Immunohistochemistry and immunofluorescence study

To investigate where human AQP5 is expressed in AQP5 transgenic mouse, multiple tissue and organ samples have been collected and immunohistochemistry and RT-PCR were performed. Out of several organs where human AQP5 is expressed, we are presenting immunohistochemistry findings of slides from salivary gland and lung tissue samples, both of sites where prior studies have shown that both human AQP5 and mice AQP5 are strongly expressed (Fig. 1B). We found that AQP5 expression was clearly detected in both organs and in both slides, there is enhanced expression of human AQP5 as compared to control (non-transgene) and overall, there is significant cellular hyperplasia in transgene over control. Also, we have performed immunofluorescence analysis for both colon and lung tissue samples (Fig. 1C). In both colon and lung tissue of AQP5 transgenic mice, there were enhanced expressions of AQP5 as compared to control and both tissues samples were filled with increased number of various kinds hyperplastic cell populations as

compared to control [7,19]. In both lung and colon tissue samples, the size of vascular wall seems to be augmented as compared to controls and overall, airway/vascular smooth muscle layer seems to be enlarged as compared to those from control mice. Lung tissue from AQP5 transgenic mice showed increase of cell numbers and intensity of AQP5 expression by immunofluorescence. Interestingly, significant number of cells with intense AQP5 expression was localized in certain areas of lung tissues, which suggests that these cell might be a preneoplastic area, leading to full cancer development.

3.3. Increased activating phosphorylation of RAF, ERK and AKT

Activation of human AQP5 mediated RAF-ERK and AKT mediated signaling pathway measured by immunoblot probed with activating phosphor antibody in low, medium high copy number transgenic mice [4,17-19]. Each of four tissue samples from lung liver kidney and salivary gland, activation of RAF ERK and AKT have been detected in copy number dependent manner (Fig. 1D).

3.4. Increased tyrosine kinase activity among transgenic mice induced by human AQP

Based on the finding from cell experiment and initial analysis showing AQP5 can activate pathways down stream of RAS or SRC [4,17-19], we have reasoned that AQP5 may carry tyrosine kinase activity like EGFR or SRC. Therefore, we have measured total tyrosine kinase activity of high copy number human AQP5 transgenic mouse 2 days after birth with their controls. The kinase activity of transgene was increased up to 11.2 percentages over controls (Fig. 2A). To estimate tissue specific tyrosine kinase activity when mice were more grown, various organs and tissues were isolated and each specific tyrosine kinase were measured from high copy number AQP5 transgenic mouse at the age of 6 month (Fig. 2B). In summary, tyrosine kinase assay has demonstrated significantly increased activity among 8 different organs including White Adipose

Tissue (WAT), Spleen, Lung, Hypothalamus, Colon, Heart, Skin, and Gastrocnemius (Fig. 2B).

3.5. Baculovirus mediated preparation of recombinant AQP5 and its mutants and their kinase activities

To directly measure tyrosine kinase activity of AQP5, we purified recombinant AQP5 (rAQP5) protein using baculovirus system. Various Flag tagged recombinant human AQP5 proteins were purified using baculovirus system and its kinase activity was measured. Flag-tagged wild type human AQP5 were harvested 72 h after infection and cell extracts were measured for tyrosine kinase activity by assay kit. Kinase activity from human AQP infected cells is significantly higher than non-transfected control and immunoblotting using anti Flag antibody was as infection control (Fig.3A). After this proof of principal experiment, next steps of amplification and purification steps for recombinant protein for wild type and two of its mutants were carried out. We have used three different human AQP5 construct as used in prior reports. A diagram of Flag-tagged wild type human AQP5 cDNA expression construct, its mutants S156A and N185D are presented (Fig. 3B). In S156A mutant, Serine 156 is replaced by Alanine and in N185D mutant, Asparagine is replaced with Aspartic acid. From prior cell line experiments, it has been demonstrated that both mutants could not activate Ras, Erk and Src as strongly as wild type AQP5and that oncogenic property of AQP5 was lost by these two mutants [4,17-19].

Extract from cells transfected with Flag-tagged wild type and its Flag tagged PKA mutants human AQP5 (S156A) in addition to Flag tagged Loop mutants human AQP5 (N185D) were measured for tyrosine kinase activity. Tyrosine kinase activity was significantly increased from recombinant human wild type AQP5 while, its mutants (PKA (N185D) and Loof (S156A)) did not show any increased kinase activities over control (Fig.3C) [3,4,5,10,13-18]. These finding suggests that PKA phosphorylation, which is compromised by S156A, as well as membranous expression

of AQP5, which is compromised by N185D, might be crucial for the tyrosine kinase activity conferred by human AQP5. However, we could not exclude these mutations may cause protein mis-folding [3,5,13]. Moreover, during the purification of rAQP5 protein, molecular weight of more than 50 kDa rAQP5 protein fraction possessed tyrosine kinase activity, while monomeric rAQP5 did not show any significant kinase activity (data not shown). This result suggested that monomeric form of rAQP5 may not form a molecular unit possessing tyrosine kinase activity.

3.6. Recombinant AQP5 augment kinase activity from EGFR protein

Purified wild type human recombinant AQP5 protein as used in Fig. 3 was incubated with recombinant EGFR proteins and kinase assay were measured. At the top of bar, an average fold induction of 3 experiments were indicated. First, in this experimental setting, wild type human rAQP5 (fold activity 3.6) has demonstrated approximately 2 times higher tyrosine kinase activity as compared to recombinant EGFR (fold activity 1.8). Moreover, when both recombinant proteins are combined, measurement of tyrosine kinase activity demonstrates a potentially synergistically augmented effect (fold activity 7.8) (Fig. 4A). However, such augmentative effects were not observed between wild type AQP5 and Src (Fig. 4B). To elucidate whether AQP5 can enhance tyrosine phosphorylation of EGFR, expression construct for EGFR and wild type AQP5 Flag constructs were co-transfected into Chinese Hamster Ovary (CHO) cell line, which does not express detectable levels of EGFR, ERBB2, or ERBB3 (28). EGFR phosphorylation (Tyr845) is increased dependent on amount of transfected AQP5 construct (Fig. 4C).

4. Discussion

Tumor cell growth largely depends on signaling from extracellular space to the intracellular space, as seen in EGF to EGFR mediated signaling pathways [20]. Likewise, cell migration is

involved in tumor growth and metastatic process [20-22]. Fundamentally, endothelial cell migration plays a key role in angiogenesis in the development of normal organs and growth of tumor cells. In the last 10 years, with following the initial report from our group for on the role of AQP during colorectal development [7] and several tumor cell types were shown to express AQPs *in vivo* in humans and rodents. Furthermore, prognostic implications have been established based on AQP expression in lung and, brain tumors and human glioblastoma [3,4,10].

In this report, we have demonstrated overall cellular hyperplasia from human AQP5 expression in transgenic mice. Moreover, we have demonstrated dose dependent activation of RAF, ERK and AKT activation. We have reasoned that activation of RAS, RAF ERK and AKT shares common downstream pathways from EGFR. We therefore measured tyrosine kinase activity from transgenic mice and demonstrated enhanced tyrosine kinase activity. Finally, by using recombinant human AQP5 protein which was amplified and purified by using known bacuolar virus system, we have demonstrated that wild type AQP5 does carry tyrosine kinase activity while two of its mutants does not. A comparison of AQP5 kinase activity with known tyrosine kinase like EGFR and SRC seems to suggest AQP5 as a molecule carrying potential tyrosine kinase activity. Moreover, a potential synergistic augmented kinase activity was observed when AQP5 and EGFR are combined. Additionally, we have demonstrated that in cell line model, the degree of EGFR tyrosine phosphorylation depends on amount of transfected human AQP5.

A gene copy number dependent, AQP5 induced activation of downstream pathways from RAS into RAF ERK and AKT pathways are expected from previous cell studies [4,17-19]. Also, transgene expression of human AQP5 is distributed among known AQP5 expression sites. Enhanced tyrosine kinase activity from transgenic mice provides an important insight toward overall impact of AQP5 protein on various cellular pathophysiology and supports the notion of

AQP5 as a potential oncogene. Tyrosine kinase assay with recombinant wild type human AQP5 and its two mutants prepared from baculovirus system is consistent with previous cell line study demonstrating that AQP5 can activate a series of common pathways induced by tyrosine kinase, namely RAS, RAF, ERK and AKT activation [17-19].

Protein kinase activation is a common mechanism of tumorigenesis [20-22,24]. Kinases regulate many aspects that control cell growth, movement, and death. Deregulated or over-activation of kinase activity is a frequent cause of various human disease, particularly cancer. Targeting tyrosine kinase like EGFR or BCR ABL overall become as a key therapeutic approach and have been widely used in the field of clinical oncology [25-27]. Inhibition of activated protein kinases using targeted small molecular drugs or antibody-based strategies overall are rapidly expanded as an effective approach to 21st century cancer therapy [21-22,24-27]. Small molecule inhibitor like Gefitinib (Iressa) and erlotinib (Tarceva) and therapeutic antibody like cetuximab inhibit the EGFR kinase have been widely used to treat non–small cell lung cancers (NSCLCs) and head and neck cancer [28-34]. The data we present here, AQP5 as a tyrosine kinase like EGFR, may provide a biological rationale for designing novel anti AQP5 therapeutics and in fact, we have initiated designing anti AQP5 therapeutic antibody. Recently, we have developed 4 novel therapeutic small molecules, which have been tested into various cancer cell lines, and demonstrated various *in vitro* therapeutic efficacy (Kang et al, manuscript in prep). We envision that an anti AQP5 therapeutic antibody or small molecular inhibitors targeted to AQP5 among patients' group with strong AQP5 expression may enter clinical in the near future [17,19,23].

There is growing evidence in several tumor types to indicate that several growth factors (e.g., EGF, VEGF and FGF), which are known to enhance cell growth, invasion, may do so, at least in part, through increasing expression of several AQPs [4,7,17-19,23]. This is likely to be mediated

by interaction with various signaling molecules such as Ras, MAPK and PI3K, all of which also mediate downstream signaling from AQP5, leading to rearrangement of the actin cytoskeleton (through interaction with RhoA/Rac), extracellular acidification (through interaction with LDH and HIF-1α) [23]. Importantly, recent report suggested that AQP5 may be a cancer stem gene responsible for gastric cancer development [23,35]. Here, our report demonstrating tyrosine kinase activity conferred by AQP5 may expand our present understanding for potential mechanism underlying AQP induced carcinogenesis. We expect to see a continued expansion of key molecular mechanistic studies leading to better understandings of aquaporin biology during human carcinogenesis.

Declaration of Competing Interests

The authors declare that they have no known competing financial interests or personal relationships that could have appeared to influence the work reported in this paper.

Authors' contributions

CM was the Principal Investigator of the grant that supported this project. CM and SK conceived of and executed the overall study and design, oversaw all molecular data collection, directed the analysis plan. CM wrote the paper. CM and SK together contributed to the analysis plan, contributed to the interpretation of the results, and approved the final manuscript.

Acknowledgements

This study was supported in part by the SPORE grant (grant number P50 CA96784-01; to C.S.M.), Cancer Research Grant from Pyung-Ya Foundation (grant number PY-1; to C.S.M.) and

Translational Research Grant from HJM Foundation (grant number HJM-TR1; to C.S.M. and D.M.). C.S.M. is deeply grateful to Dr. H.J. Moon for incessant support for this study.

References

[1] L.S. King, P. Agre, Pathophysiology of the aquaporin water channels, Annu. Rev. Physiol. 58 (1996) 619–648. https://doi.org/10.1146/annurev.ph.58.030196.003155.

[2] A.S. Verkman, More than just water channels: unexpected cellular roles of aquaporins, J. Cell Sci. 118 (2005) 3225–3232. https://doi.org/10.1242/jcs.02519.

[3] S. Saadoun, M.C. Papadopoulos, D.C. Davies, S. Krishna, B.A. Bell, Aquaporin-4 expression is increased in oedematous human brain tumours, J. Neurol. Neurosurg. Psychiatry 72 (2002) 262–265. https://doi.org/10.1136/jnnp.72.2.262.

[4] J. Woo, J. Lee, M.S. Kim, S.J. Jang, D. Sidransky, C. Moon, The effect of aquaporin 5 overexpression on the Ras signaling pathway, Biochem. Biophys. Res. Commun. 367 (2008) 291–298. https://doi.org/10.1016/j.bbrc.2007.12.073.

[5] A. Warth, P. Simon, D. Capper, B. Goeppert, G. Tabatabai, H. Herzog, K. Dietz, F. Stubenvoll, R. Ajaaj, R. Becker, M. Weller, R. Meyermann, H. Wolburg, M. Mittelbronn, Expression pattern of the water channel aquaporin-4 in human gliomas is associated with blood–brain barrier disturbance but not with patient survival, J. Neurosci. Res. 85 (2007) 1336–1346. https://doi.org/10.1002/jnr.21224.

[6] M. Endo, R.K. Jain, B. Witwer, D. Brown, Water channel (aquaporin 1) expression and distribution in mammary carcinomas and glioblastomas, Microvasc. Res. 58 (1999) 89–98. https://doi.org/10.1006/mvre.1999.2158.

[7] C. Moon, J.C. Soria, S.J. Jang, J. Lee, M.O. Hoque, M. Sibony, B. Trink, Y.S. Chang, D.

Sidransky, L. Mao, Involvement of aquaporins in colorectal carcinogenesis, Oncogene 22 (2003) 6699–6703. https://doi.org/10.1038/sj.onc.1206762.

[8] S. Saadoun, M.C. Papadopoulos, D.C. Davies, B.A. Bell, S. Krishna, Increased aquaporin 1 water channel expression in human brain tumours, Br. J. Cancer 87 (2002) 621–623. https://doi.org/10.1038/sj.bjc.6600512.

[9] Y. Chen, O. Tachibana, M. Oda, R. Xu, J.I. Hamada, J. Yamashita, N. Hashimoto, J.A. Takahashi, Increased expression of aquaporin 1 in human hemangioblastomas and its correlation with cyst formation, J. Neurooncol. 80 (2006) 219–225. https://doi.org/10.1007/s11060-005-9057-1.

[10] A. Vacca, A. Frigeri, D. Ribatti, G.P. Nicchia, B. Nico, R. Ria, M. Svelto, F. Dammacco, Microvessel overexpression of aquaporin 1 parallels bone marrow angiogenesis in patients with active multiple myeloma, Br. J. Haematol. 113 (2001) 415–421. https://doi.org/10.1046/j.1365-2141.2001.02738.x.

[11] M.O. Hoque, J.C. Soria, J. Woo, T. Lee, J. Lee, S.J. Jang, S. Upadhyay, B. Trink, C. Monitto, C. Desmaze, L. Mao, D. Sidransky, C. Moon, Aquaporin 1 is overexpressed in lung cancer and stimulates NIH-3T3 cell proliferation and anchorage-independent growth, Am. J. Pathol. 168 (2006) 1345–1353. https://doi.org/10.2353/ajpath.2006.050596.

[12] S. Saadoun, M.C. Papadopoulos, M. Hara-Chikuma, A.S. Verkman, Impairment of angiogenesis and cell migration by targeted aquaporin-1 gene disruption, Nature 434 (2005) 786–792. https://doi.org/10.1038/nature03460.

[13] J. Hu, A.S. Verkman, Increased migration and metastatic potential of tumor cells expressing aquaporin water channels, FASEB J. 20 (2006) 1892–1894. https://doi.org/10.1096/fj.06-5930fje.

[14] M. Hara-Chikuma, A.S. Verkman, Aquaporin-1 facilitates epithelial cell migration in kidney proximal tubule, J. Am. Soc. Nephrol. 17 (2006) 39–45. https://doi.org/10.1681/asn.2005080846.

[15] K.I. Auguste, S. Jin, K. Uchida, D. Yan, G.T. Manley, M.C. Papadopoulos, A.S. Verkman, Greatly impaired migration of implanted aquaporin-4-deficient astroglial cells in mouse brain toward a site of injury, FASEB J. 21 (2007) 108–116. https://doi.org/10.1096/fj.06-6848com.

[16] B. Burghardt, M.L. Elkaer, T.H. Kwon, G.Z. Rácz, G. Varga, M.C. Steward, S. Nielsen, Distribution of aquaporin water channels AQP1 and AQP5 in the ductal system of the human pancreas, Gut 52 (2003) 1008–1016. https://doi.org/10.1136/gut.52.7.1008.

[17] S.K. Kang, Y.K. Chae, J. Woo, M.S. Kim, J.C. Park, J. Lee, J.C. Soria, S.J. Jang, D. Sidransky, C. Moon, Role of human aquaporin 5 in colorectal carcinogenesis, Am. J. Pathol. 173 (2008) 518–525. https://doi.org/10.2353/ajpath.2008.071198.

[18] J. Woo, J. Lee, Y.K. Chae, M.S. Kim, J.H. Baek, J.C. Park, M.J. Park, I.M. Smith, B. Trink, E. Ratovitski, T. Lee, B. Park, S.J. Jang, J.C. Soria, J.A. Califano, D. Sidransky, C. Moon, Overexpression of AQP5, a putative oncogene, promotes cell growth and transformation, Cancer Lett. 264 (2008) 54–62. https://doi.org/10.1016/j.canlet.2008.01.029.

[19] Y.K. Chae, J. Woo, M.J. Kim, S.K. Kang, M.S. Kim, J. Lee, S.K. Lee, G. Gong, Y.H. Kim, J.C. Soria, S.J. Jang, D. Sidransky, C. Moon, Expression of aquaporin 5 (AQP5) promotes tumor invasion in human non small cell lung cancer, PLoS One 3 (2008) e2162. https://doi.org/10.1371/journal.pone.0002162.

[20] M. Okoye, G. Sexton G, E. Huang, J. McCaffery, P. Desai . Functional analysis of the triplex proteins (VP19C and VP23) of herpes simplex virus type 1. J Virol.

2006;80(2):929-940. doi:10.1128/JVI.80.2.929-940.2006.

[21] M. Scaltriti, J. Baselga, The epidermal growth factor receptor pathway: a model for targeted therapy, Clin. Cancer Res. 12 (2006) 5268–5272. https://doi.org/10.1158/1078-0432.ccr-05-1554.

[22] N. Prenzel, E. Zwick, H. Daub, M. Leserer, R. Abraham, C. Wallasch, A. Ullrich, EGF receptor transactivation by G-protein-coupled receptors requires metalloproteinase cleavage of proHB-EGF, Nature 402 (1999) 884–888. https://doi.org/10.1038/47260.

[22] G. Manning, D.B. Whyte, R. Martinez, T. Hunter, S. Sudarsanam, The protein kinase complement of the human genome, Science 298 (2002) 1912–1934. https://doi.org/10.1126/science.1075762.

[23] C. Moon, D. Moon, Aquaporins in carcinogenesis: water and glycerol channels as new potential drug targets, in: G. Soveral, S. Nielsen, A. Casini, (Eds.), Aquaporins in Health and Disease: New Molecular Targets for Drug Discovery, CRC Press, 2016. pp. 217–232. https://doi.org/10.1201/b19017-13.

[24] M. Hojjat-Farsangi, Small-molecule inhibitors of the receptor tyrosine kinases: promising tools for targeted cancer therapies, Int. J. Mol. Sci. 15 (2014) 13768–13801. https://doi.org/10.3390/ijms150813768.

[25] P.W. Manley, S.W. Cowan-Jacob, E. Buchdunger, D. Fabbro, G. Fendrich, P. Furet, T. Meyer, J. Zimmermann, Imatinib: a selective tyrosine kinase inhibitor, Eur. J. Cancer 38 (2002) S19–S27. https://doi.org/10.1016/s0959-8049(02)80599-8.

[26] C.L. Arteaga, ErbB-targeted therapeutic approaches in human cancer, Exp. Cell Res. 284 (2003) 122–130. https://doi.org/10.1016/s0014-4827(02)00104-0.

[27] B.J. Druker, M. Talpaz, D.J. Resta, B. Peng, E. Buchdunger, J.M. Ford, N.B. Lydon, H.

Kantarjian, R. Capdeville, S. Ohno-Jones, C.L. Sawyers, Efficacy and safety of a specific inhibitor of the BCR-ABL tyrosine kinase in chronic myeloid leukemia, N. Engl. J. Med. 344 (2001) 1031–1037. https://doi.org/10.1056/nejm200104053441401.

[28] M.G. Kris, R.B. Natale, R.S. Herbst, J.T.J. Lynch, D. Prager, C.P. Belani, J.H. Schiller, K. Kelly, H. Spiridonidis, A. Sandler, K.S. Albain, D. Cella, M.K. Wolf, S.D. Averbuch, J.J. Ochs, A.C. Kay, Efficacy of gefitinib, an inhibitor of the epidermal growth factor receptor tyrosine kinase, in symptomatic patients with non–small cell lung cancer: a randomized trial, JAMA 290 (2003) 2149–2158. https://doi.org/10.1001/jama.290.16.2149.

[29] C. Moon, Y.K. Chae, J. Lee, Targeting epidermal growth factor receptor in head and neck cancer: lessons learned from cetuximab, Exp. Biol. Med. 235 (2010) 907–920. https://doi.org/10.1258/ebm.2009.009181.

[30] J.G. Paez, P.A. Jänne, J.C. Lee, S. Tracy, H. Greulich, S. Gabriel, P. Herman, F.J. Kaye, N. Lindeman, T.J. Boggon, K. Naoki, H. Sasaki, Y. Fujii, M.J. Eck, W.R. Sellers, B.E. Johnson, M. Meyerson, EGFR mutations in lung cancer: correlation with clinical response to gefitinib therapy, Science 304 (2004) 1497–1500. https://doi.org/10.1126/science.1099314.

[31] T.J. Lynch, D.W. Bell, R. Sordella, S. Gurubhagavatula, R.A. Okimoto, B.W. Brannigan, P.L. Harris, S.M. Haserlat, J.G. Supko, F.G. Haluska, D.N. Louis, D.C. Christiani, J. Settleman, D.A. Haber, Activating mutations in the epidermal growth factor receptor underlying responsiveness of non–small-cell lung cancer to gefitinib, N. Engl. J. Med. 350 (2004) 2129–2139. https://doi.org/10.1056/nejmoa040938.

[32] W. Pao, V. Miller, M. Zakowski, J. Doherty, K. Politi, I. Sarkaria, B. Singh, R. Heelan, V. Rusch, L. Fulton, E. Mardis, D. Kupfer, R. Wilson, M. Kris, H. Varmus, EGF receptor gene

mutations are common in lung cancers from "never smokers" and are associated with sensitivity of tumors to gefitinib and erlotinib, Proc. Natl. Acad. Sci. U. S. A. 101 (2004) 13306–13311. https://doi.org/10.1073/pnas.0405220101.

[33] W. Pao, V.A. Miller, Epidermal growth factor receptor mutations, small-molecule kinase inhibitors, and non–small-cell lung cancer: current knowledge and future directions, J. Clin. Oncol. 23 (2005) 2556–2568. https://doi.org/10.1200/jco.2005.07.799.

[34] G. Giaccone, J.A. Rodriguez, EGFR inhibitors: what have we learned from the treatment of lung cancer?, Nat. Clin. Pract. Oncol. 2 (2005) 554–561. https://doi.org/10.1038/ncponc0341.

[35] Tan, S.H., Swathi, Y., Tan, S. et al. AQP5 enriches for stem cells and cancer origins in the distal stomach. Nature 578, 437–443 (2020). https://doi.org/10.1038/s41586-020-1973

Figure Legends

Fig. 1. Generation of transgenic mice. (A) Three lines of transgenic mice were generated and various organs from mice carrying low copy (1), medium copy (2) and high copy number (3) of human AQP5 transgene are harvested. Different copy number of genomic DNA (DNA) and messenger RNA (cDNA) were determined by PCR amplification with GAPDH expression as internal control. (B) Immunohistochemistry analysis showed clear AQP5 expression in lung and salivary grand. (C) Immunofluorescence of human AQP5 expression in lung and colon tissues between tissues carrying AQP5 transgene and its control. Arrows indicate abundant expression of AQP5 by immunofluorescence using anti-goat AQP5 antibody in colon and lung tissues in high copy number AQP5 transgenic mouse. (D) Activation of human AQP5 transgene mediated RAF-ERK and AKTsignaling pathway measured by activating phosphor antibody in low, medium high copy number transgenic mice. Total protein level of RAF, ERK and AKT are shown as internal control.

Fig. 2. AQP5 activates kinase activities in AQP5 transgenic mouse. (A) Total proteins (25 μg) extracted from AQP5 transgenic and control B6SJLF1 mouse (sacrificed 2 days after born) were subjected into measurement of kinase activity using Perkin Elmer kinase assay kit. (B) Kinase activities from several tissues described in this figure were compared among AQP5 transgenic and control B6SJLF1 mice (sacrificed at 6 months after born). For both A and B, an average of 6 controls (3 male and 3 female) and 6 high copy number transgenic mice (3 male and 3 female) were used. AQP, aquaporin water channel. In the organs marked with star are those with significantly increased kinase activity by AQP5 transgene.

Fig. 3. Baculovirus mediated preparation of recombinant AQP5 and its mutants with measurement of their kinase activities. Various Flag tagged recombinant human AQP5 proteins were purified

using baculovirus system and its kinase activity was measured. (A) Flag-tagged wild type human AQP5 were harvested 72 h after infection and cell extracts were measured for tyrosine kinase activity. Kinase activities are shown both control constructed and wild type human AQP5 construct as unit activity based on manufacture guideline as in material and method. Immunoblotting using anti Flag antibody is shown as infection control. (B) Diagram of Flag-tagged wild type human AQP5 cDNA expression construct, its mutants S156A and N185D are presented. In S156A mutant, Serine 156 is replaced by Alanine and in N185D mutant, Asparagine is replaced with Aspartic acid. (C) Purified recombinant Flag-tagged wild type and its Flag tagged PKA mutant (S156A) human AQP5 in addition to Flag tagged Loop mutant (N185D) human AQP5 were measured for tyrosine kinase activity. Kinase activities are shown both control (kinase activity of extract from non-transfected cells), AQP5 (kinase activity of extract from Flag tagged wild type human AQP5 construct), PKA (kinase activity of extract from Flag tagged human PKA mutant AQP5 construct) and Loof (kinase activity of extract from Flag tagged human Loof mutant AQP5). Immunoblots using anti Flag antibody as internal control were shown at the bottom of assay graph. AQP, aquaporin water channel; PKA, cAMP-protein kinase.

Fig. 4. Recombinant AQP5 augment kinase activity from EGFR. Purified wild type human recombinant AQP5 protein as used in Fig. 3 was incubated with recombinant EGFR proteins and kinase assay were measured (A). At the top of bar, an average fold induction of 3 experiments were indicated. Wild type human rAQP5 (fold activity 3.6) and recombinant EGFR (fold activity 1.8), when both are combined together, shows augmented protein kinase activity (fold activity 7.8) (A). However, such augmentative effects were not observed when AQP5 protein is combined with Src (B). EGFR null cell line, CHO cells were transiently transfected with EGFR (1 ug) and increasing amount of AQP5 expression plasmid (0.01, 0.05, 0.1, and 0.5 ug of Flag-AQP5). In each amount

of AQP5 expression plasmid transfection, expressions of EGFR and amount of pEGFR (Tyr 845) were measured by immunoblotting (C). AQP, aquaporin water channel; EGFR, epidermal growth factor receptor; pEGFR (Tyr 845), Tyrosine phosphorylation at amino acid 845 of EGFR protein; rAQP5, recombinant AQP5.
